# Insulin-Like Growth Factor Binding Protein-6 Promotes the Differentiation of Placental Mesenchymal Stem Cells into Skeletal Muscle Independent of Insulin-Like Growth Factor Receptor-1 and Insulin Receptor

**DOI:** 10.1155/2019/9245938

**Published:** 2019-02-17

**Authors:** Doaa Aboalola, Victor K. M. Han

**Affiliations:** ^1^Western University, Department of Anatomy and Cell Biology, Schulich School of Medicine and Dentistry, London, Ontario, Canada; ^2^Western University, Children's Health Research Institute, London, Ontario, Canada; ^3^Western University, Lawson Health Research Institute, London, Ontario, Canada; ^4^King Abdullah International Medical Research Center, Jeddah, Saudi Arabia; ^5^King Saud bin Abdulaziz University for Health Sciences, Jeddah, Saudi Arabia; ^6^Ministry of National Guard Health Affairs, Jeddah, Saudi Arabia; ^7^Western University, Department of Paediatrics, Schulich School of Medicine and Dentistry, London, Ontario, Canada

## Abstract

As mesenchymal stem cells (MSCs) are being investigated for regenerative therapies to be used in the clinic, delineating the roles of the IGF system in MSC growth and differentiation, *in vitro*, is vital in developing these cellular therapies to treat degenerative diseases. Muscle differentiation is a multistep process, starting with commitment to the muscle lineage and ending with the formation of multinucleated fibers. Insulin-like growth factor binding protein-6 (IGFBP-6), relative to other IGFBPs, has high affinity for IGF-2. However, the role of IGFBP-6 in muscle development has not been clearly defined. Our previous studies showed that *in vitro* extracellular IGFBP-6 increased myogenesis in early stages and could enhance the muscle differentiation process in the absence of IGF-2. In this study, we identified the signal transduction mechanisms of IGFBP-6 on muscle differentiation by placental mesenchymal stem cells (PMSCs). We showed that muscle differentiation required activation of both AKT and MAPK pathways. Interestingly, we demonstrated that IGFBP-6 could compensate for IGF-2 loss and help enhance the muscle differentiation process by triggering predominantly the MAPK pathway independent of activating either IGF-1R or the insulin receptor (IR). These findings indicate the complex interactions between IGFBP-6 and IGFs in PMSC differentiation into the skeletal muscle and that the IGF signaling axis, specifically involving IGFBP-6, is important in muscle differentiation. Moreover, although the major role of IGFBP-6 is IGF-2 inhibition, it is not necessarily the case that IGFBP-6 is the main modulator of IGF-2.

## 1. Introduction

Skeletal muscle comprises one-half of the human body [1]. The development of skeletal muscle is a complex multistep process, starting with the generation of myogenic precursors from mesodermal stem cells and ending with terminal differentiation and the commitment of myoblasts into myofibers [2]. During myogenesis, muscle stem cells commit to the muscle lineage by upregulating muscle commitment markers (Pax3/7). As Pax3/7 subsequently decreases, early muscle differentiation markers (MyoD and Myogenin) begin to be expressed [3]. The committed muscle cells then start to fuse and form multinucleated fibers, which express the late muscle differentiation marker, myosin heavy chain (MHC) [3]. During muscle repair, a similar process is thought to occur whereby satellite cells become activated, migrate towards injured muscle, and begin the differentiation process to replace injured myofibers [4].

IGFs are important components of the skeletal muscle microenvironment and are required for muscle growth during development and regeneration after injury [1, 5, 6]. IGFs regulate MyoD and Myogenin gene expressions, but the mechanism is not completely understood [1]. When mice are injected with IGF-1, there is an enhancement in muscle mass (hypertrophy) [7, 8]. Moreover, IGF-1R null mice show profound muscle hypoplasia and die prematurely soon after birth due to breathing difficulties resulting from atrophy of diaphragm and respiratory muscles [9].

Following the binding of IGFs to IGF-1R or IR, IRS-1 and IRS-2 are phosphorylated, and then PI3K-AKT-mTOR and MAPK pathways are activated [10]. Therefore, crosstalk between the different receptor tyrosine kinase (RTK) pathways can lead to different cellular responses and signaling outcomes. Also, the presence of target effectors and the timing of their activation are important in determining cell fate decisions towards proliferation or differentiation [11].

During muscle differentiation, MAPK signals play an important role [12]. Marshall reported that a prolonged activation of ERK1/2 leads to differentiation, whereas a transient activation of ERK1/2 leads to proliferation, as it is not sufficient to elevate the levels of nuclear ERK1/2 [13]. Therefore, the availability of growth factors in the microenvironment and the receptors they activate determine stem cell fate through the signaling intermediates activated. Furthermore, it is known that IGFs mediate and induce myogenesis by directly activating the myogenin gene promoter. However, when the PI3K inhibitor, LY294002, which acts upstream of AKT signaling, is introduced, IGF is no longer able to induce myogenesis or enhance the expression of myogenin [14]. Therefore, the direct effects of the IGF stimulation on the myogenin promoter are also mediated via the actions of PI3K *via* AKT signaling. Additionally, IGF-1R signaling through PI3K was shown to upregulate myogenin expression leading to an enhanced myogenesis [14] and also regulate basal levels of IGF-1 and IGF-2 genes during myogenesis [14, 15]. C2BP5 myoblast differentiation was still achieved when transfected by recombinant adenoviruses expressing MyoD in the absence of IGFs [16]. When MyoD-transfected C2BP5 cells were treated with LY294002, the transcriptional activity of MyoD, Myogenin, and MHC was not inhibited but the myofibers were smaller and thinner with fewer nuclei [16]. Collectively, these studies suggested that IGF-activated PI3K-AKT and MAPK pathways are both important for myoblast differentiation [17].

IGFs interact with insulin for metabolism, survival, proliferation, and differentiation of many cell types either through IGF-1R or the insulin receptor (IR) or the IGF-1R-IR hybrid receptor [18–20]. Both the IGF-1R and IR are tyrosine protein kinases that activate multiple signaling transduction pathways [20, 21]. The PI3K-AKT pathway but not the MAPK is activated by insulin [21]. It is known that each ligand binds to its respective receptor with higher affinity and to the other receptor or hybrid receptors with lower affinity. While IGFs play a major role in cellular proliferation, differentiation, and survival, and insulin has a major role in metabolism, their functions are interchangeable depending on the concentration of the peptide in the extracellular space.

Circulating IGFs are bound to six soluble IGF-binding proteins (IGFBPs 1–6), which determine the bioavailability of free IGFs in the extracellular environment, thus modifying the IGF actions [22]. Under normal physiological conditions, IGFs bind IGFBPs with greater affinity than they bind IGF-1R, playing an important role in IGF-regulated cell metabolism, development, and growth. In addition, it has become apparent that the IGFBPs can be expressed and maintained within the cellular microenvironment and have additional functions independent of regulating IGFs [22].

In RD rhabdomyosarcoma and LIM 1215 colon cancer cells, mutant IGFBP-6 that does not bind to IGF-2 induces cellular migration, suggesting an IGF-independent function of IGFBP-6 [23]. Inhibition of ERK1/2 but not AKT impeded cellular migration [23]. We have previously reported that IGFBP-6, which has high affinity to IGF-2 [24, 25], stimulates a multipotent profile and an early commitment to the muscle lineage in PMSCs [26]. Furthermore, the impact of extracellular IGFBP-6 and silencing of endogenous IGFBP-6 suggest that the biologic actions of IGFBP-6 occur in both IGF-dependent and IGF-independent mechanisms [19, 27–29]. The mechanisms of IGF-dependent and IGF-independent actions are not yet delineated. In this study, we demonstrated that the biologic actions of IGFBP-6 on PMSC differentiation into the skeletal muscle occur independently of either IGFs or insulin signaling through IGF-1R or IR.

## 2. Materials and Methods

### 2.1. Isolation of PMSCs

PMSC isolation and experiments were conducted in accordance with the approval from the Health Sciences Research Ethics Board of Western University. Informed consent was obtained from healthy women undergoing therapeutic termination of pregnancy, and the PMSCs used in this study were isolated from 15 weeks preterm placental tissues. After surgery, chorionic villi were dissected, washed, minced with surgical scissors and forceps, and subjected to enzymatic digestion with collagenase IV (369 IU/mg), hyaluronidase (999 IU/mg) (Sigma-Aldrich), and DNase I (2,000 IU/mg) (Hoffmann-La Roche) for 10 minutes at room temperature, followed by 0.05% trypsin (Gibco/Invitrogen) for 5 minutes at room temperature. The sample was then washed for 10 minutes with 10% FBS in DMEM/F12, and the resulting single cell suspension was separated by density centrifugation over a Percoll gradient using a modified protocol by Worton et al. [26, 30].

### 2.2. Muscle Differentiation and Treatments

Cells were plated in muscle growth media (fetal bovine serum 0.05 mL/mL, fetuin 50 *μ*g/mL, epidermal growth factor 10 ng/mL, basic fibroblast growth factor 1 ng/mL, insulin 10 *μ*g/mL, and dexamethasone 0.4 *μ*g/mL) for 48 hours before changing to skeletal muscle differentiation media, which is a proprietary serum-free medium containing 10 *μ*g/mL insulin (PromoCell) for 14 days. PMSCs were treated every 3 days with 200 nM of IGF-IR inhibitor PPP, 25 *μ*M of AKT inhibitor LY294002, 10 *μ*M of MEK1/2 inhibitor U0126, or 10 *μ*M of IR inhibitor HNMPA (Santa Cruz Biotechnology) under muscle differentiation conditions. Treatment concentrations for LY294002, U0126, and HNMPA were determined by a dose-response experiment using PMSCs in muscle differentiation media (Supplementary Figure 1). For IGFBP-6 supplementation with the inhibitors, recombinant human IGFBP-6 (ProSpec) was added to the media (375 ng/mL) every 3 days at the time of media change. The dose of IGFBP-6 was based on our previous studies [26, 31].

### 2.3. Immunoblotting

Cell lysates containing 20 *μ*g of protein were added to 6x SDS gel loading buffer. Samples were resolved by molecular weight using 10% SDS-polyacrylamide gels transferred onto polyvinylidene fluoride (PVDF) membranes using Trans-Blot Turbo (Bio-Rad) with an optimized protocol depending on protein size. Membranes were blocked with 5% nonfat dry milk, gently shaking for 1 hour at room temperature in Tris-HCl buffer saline pH 8.0 with 0.1% Tween-20 (TBS-T). Blots were washed with TBS-T followed by incubation at 4°C overnight with specific primary antibodies in 5% BSA or 5% nonfat dry milk in TBS-T following the manufacturer's protocol. To detect markers of cell potency, antibodies for OCT4 antibody (Santa Cruz Biotechnology) and SOX2 (Epitomics) were used. To detect the markers of muscle differentiation, Pax3/7, MyoD, Myogenin, and Myosin heavy chain (Santa Cruz Biotechnology) were used. To detect the activated signaling molecules, we used phospho-p44/42 MAPK, p44/42 MAPK, phospho-AKT, and AKT (Cell Signaling Technology). Then membranes were washed and incubated at room temperature with the corresponding secondary HRP-conjugated antibody. Resolved protein bands were detected using chemiluminescence, and images were taken using the VersaDoc Imager (Bio-Rad) [25, 30].

### 2.4. Quantification of IGFBP-6 and IGF-2 by Enzyme-Linked Immunosorbent Assay (ELISA)

Human IGFBP-6 (RayBiotech®) and IGF-2 (ALPCO) ELISA kits were used to measure the amount of IGFBP-6 and IGF-2 secreted into PMSC-conditioned media. Standards and samples were loaded into the wells, and the immobilized antibody bound the IGFBP-6 or IGF-2 present in the sample. The wells were washed and biotinylated anti-human antibody was added. After washing, HRP-conjugated streptavidin was added; then, a TMB substrate solution was used to develop a blue color in proportion to the amount of IGFBP-6 or IGF-2 bound. The stop solution changes color from blue to yellow, and the intensity was measured at 450 nm using the Multiskan Ascent plate reader and analysis software [26, 31].

### 2.5. Aldehyde Dehydrogenase (ALDH) Activity

ALDH activity, a conserved progenitor cell function, was assessed by flow cytometry at days 1, 3, 7, and 14 using the Aldefluor™ assay (STEMCELL Technologies), as per the manufacturer's instructions. Briefly, 5 *μ*L of activated Aldefluor reagent was added to 1 mL of cell suspension and incubated for 45 minutes at 37°C. Cells were washed and resuspended in 500 *μ*L of ice-cold Aldefluor assay buffer, and ALDH activity was measured using flow cytometry. As a negative control, Aldefluor™ DEAB reagent was used [26, 31].

### 2.6. Statistical Analysis

All experiments were performed in triplicate from one 15-week placental tissue (technical replicates). GraphPad Prism Software 5.0 was used to generate all graphs and analyses. A two-way ANOVA followed by Bonferroni's multiple comparison test or a one-way ANOVA followed by Student's *t*-test was used to calculate significant differences when *P* < 0.05. Graphic representation values are presented as mean ± SEM (shown as variance bars).

## 3. Results

### 3.1. IGF-1R and IGFBP-6 Are Required for PMSC Differentiation into the Skeletal Muscle

**Figure 1:**
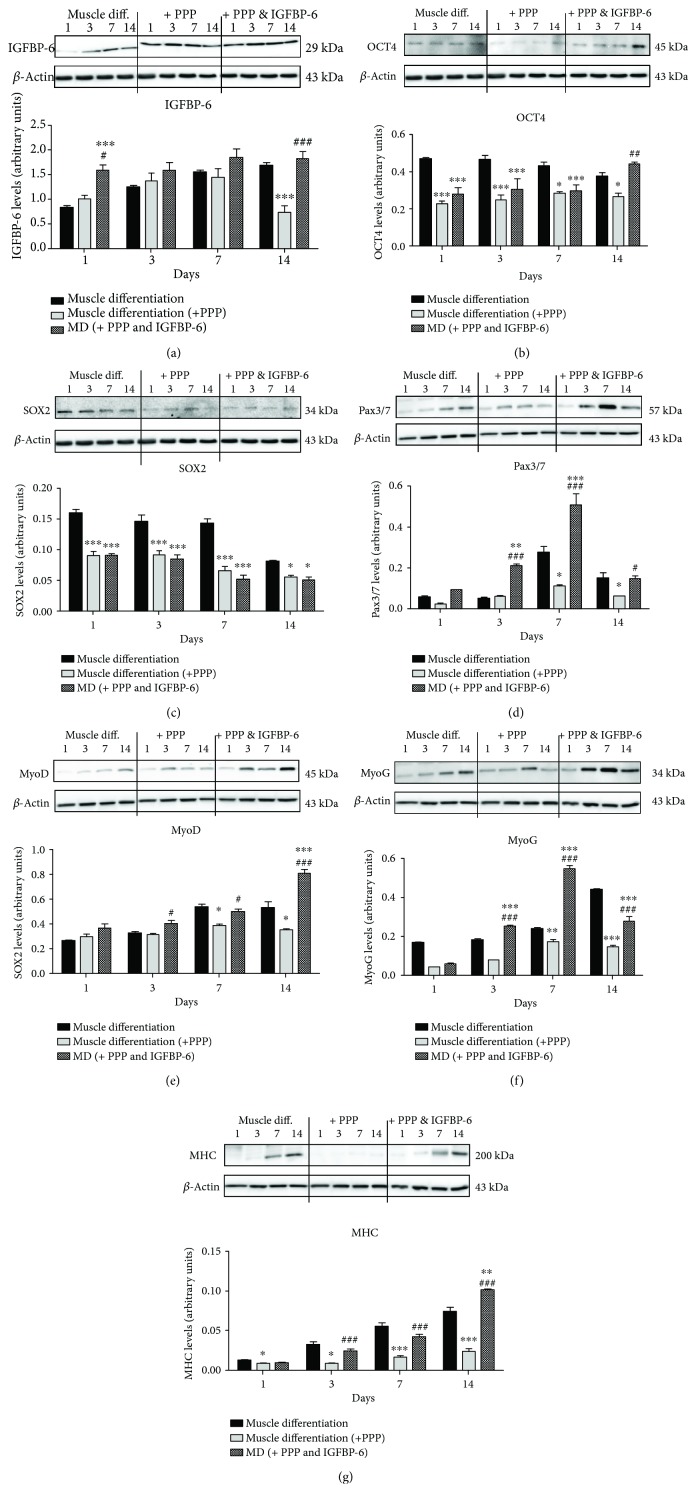
PMSCs treated with the IGF-1R inhibitor, PPP, decreased potency-associated and muscle differentiation markers. (a) PPP treatment decreased IGFBP-6 protein levels at day 14 as compared to the PMSCs grown in muscle differentiation media only. IGFBP-6 supplementation with PPP increased IGFBP-6 levels at 1 and 14 days compared to PPP alone. PPP treatment also decreased the protein levels of the potency-associated markers (b) OCT4 and (c) SOX2. When IGFBP-6 was added with PPP, OCT4 levels increased at 14 days. (d) PPP treatment decreased the protein levels of muscle lineage commitment marker Pax3/7 at 7 and 14 days. IGFBP-6 supplementation with PPP increased Pax3/7 from 3 to 14 days compared to PPP alone. (e, f) Levels of the muscle differentiation markers, MyoD and MyoG, were decreased at 7 and 14 days, and adding IGFBP-6 with PPP reversed these effects. (g) Conversely, MHC protein levels were reduced with PPP treatment at all time points compared to muscle differentiation. IGFBP-6 supplementation with PPP increased MHC levels from 3 to 14 days compared to PPP alone. Protein levels were quantified by densitometry and normalized to *β*-actin. Data is presented as the mean ± SEM of 3 independent experiments from one preterm placenta. Two-way ANOVA with Bonferroni's multiple comparison test was performed to determine ^∗^ *P* < 0.05, ^∗∗^ *P* < 0.01, and ^∗∗∗^ *P* < 0.001 compared to muscle differentiation conditions or ^#^ *P* < 0.05, ^##^ *P* < 0.01, and ^###^ *P* < 0.001 compared to PPP.

To evaluate the effects of IGF-1R inhibition on potency-associated and muscle differentiation markers in PMSCs under muscle differentiation conditions, PPP (IGF-1R-specific autophosphorylation inhibitor) was used during PMSC muscle differentiation for 14 days with/without IGFBP-6 supplementation every 3 days. As determined by immunoblotting, the presence of PPP decreased IGFBP-6 protein levels at 14 days (Figure 1(a)). Potency-associated marker (OCT4 and SOX2) levels were decreased by PPP treatment compared to muscle differentiation alone (Figures 1(b) and 1(c)). The muscle commitment marker Pax3/7 levels were decreased by PPP treatment at 7 and 14 days (Figure 1(d)). Similarly, the protein levels of the muscle lineage differentiation markers MyoD and MyoG were decreased at 7 and 14 days (Figures 1(e) and 1(f)). In contrast, MHC levels were reduced at all time points after PPP treatment compared to muscle differentiation (Figure 1(g)). Overall, PPP treatment significantly delayed muscle lineage commitment and differentiation *in vitro*.

To determine whether IGFBP-6 could rescue PMSC differentiation into the skeletal muscle during IGF-1R inhibition, extracellular IGFBP-6 was added to the culture alongside PPP supplementation. As predicted, IGFBP-6 levels increased after coadministration of IGFBP-6 with PPP at day 14 compared to the inhibitor alone (Figure 1(a)). Also, OCT4 protein levels were increased at 14 days with the combined treatments, while SOX2 levels were not changed compared to the inhibitor alone (Figures 1(b) and 1(c)). Furthermore, IGFBP-6 supplementation with PPP increased the levels of the muscle lineage differentiation markers Pax3/7, MyoD, MyoG, and MHC from 3 to 14 days compared to PPP alone (Figures 1(d)–1(g)). Moreover, the addition of IGFBP-6 alone without PPP was tested, and there were no significant changes compared to the addition of IGFBP-6 with PPP (data not shown). These findings indicate that IGFBP-6 may be an important regulator of skeletal muscle differentiation and its action, in part, occurred without activating IGF-1R signaling and independent of IGF.

**Figure 2:**
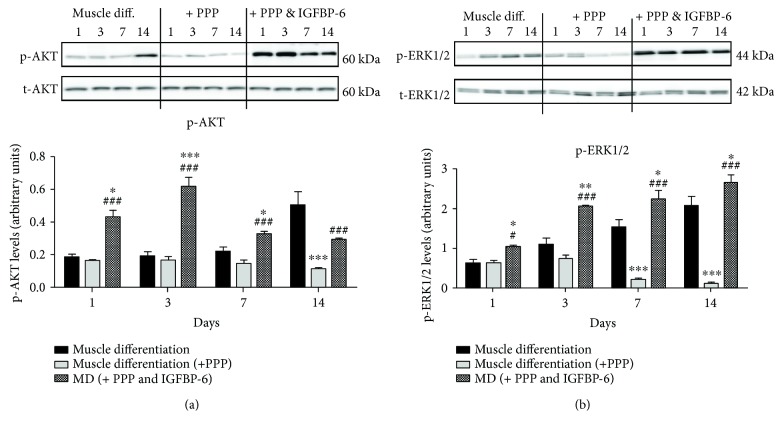
PMSCs treated with IGF-1R inhibitor (PPP) showed reduced p-AKT and p-ERK1/2 levels that were reversed by IGFBP-6 addition. (a, b) PMSCs treated with PPP showed lower protein levels of p-AKT p-ERK1/2 at the later time points when compared to the PMSCs under muscle differentiation conditions alone. When IGFBP-6 was added to PPP, p-AKT and p-ERK1/2 levels increased at all time points compared to PPP alone. Protein levels were quantified by densitometry and normalized to total AKT or total ERK1/2. Data is presented as the mean ± SEM of 3 independent experiments from one preterm placenta. Two-way ANOVA with Bonferroni's multiple comparison test was performed to determine ^∗^ *P* < 0.05, ^∗∗^ *P* < 0.01, and ^∗∗∗^ *P* < 0.001 compared to muscle differentiation conditions or ^#^ *P* < 0.05 and ^###^ *P* < 0.001 compared to PPP.

**Figure 3:**
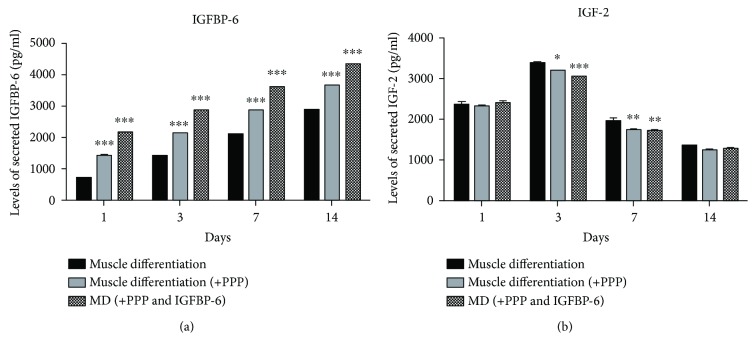
PMSCs treated with PPP showed increased IGFBP-6 secretion but decreased IGF-2 secretion. (a) IGFBP-6 secretion was increased after PPP treatment at all time points compared to muscle differentiation conditions alone. (b) IGF-2 secretion was reduced at 3 and 7 days compared to muscle differentiation conditions alone. IGFBP-6 with PPP did not have an additional effect. Data is presented as the mean ± SEM of 3 independent experiments from one preterm placenta. Two-way ANOVA with Bonferroni's multiple comparison test was performed to determine ^∗^ *P* < 0.05, ^∗∗^ *P* < 0.01, and ^∗∗∗^ *P* < 0.001 compared to muscle differentiation conditions alone.

Downstream of the IGF-1R signaling, the presence of PPP during muscle differentiation caused a reduction in p-AKT levels at 7 days which was significant by day 14 and in p-ERK1/2 levels at 7 and 14 days when compared to muscle differentiation alone (Figures 2(a) and 2(b)). In contrast, IGFBP-6 increased both p-AKT and p-ERK1/2 protein levels at all time points in the presence of PPP under muscle differentiation conditions compared to PPP alone indicating that IGFBP-6 may trigger MAPK signal transduction cascade independent of IGFs (Figures 2(a) and 2(b)). In the presence of PPP, IGFBP-6 secretion into the conditioned media was increased compared to muscle differentiation (Figure 3(a)), whereas IGF-2 secretion was reduced at days 3 and 7 (Figures 3(b)); these effects could be because the exogenously added IGFBP-6 is internalized (data not shown).

### 3.2. IGFBP-6 Is Required for PMSC Muscle Differentiation after Inhibition of the PI3K Pathway

**Figure 4:**
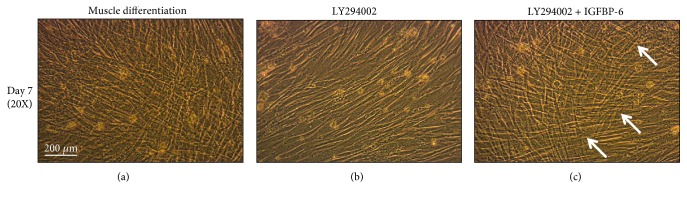
PMSCs treated with LY294002 reduced differentiated muscle compaction at 7 and 14 days, while IGFBP-6 with LY294002 delayed muscle compaction changes to 14 days. Higher magnification of PMSCs treated with LY294002 or with IGFBP-6 supplementation with LY294002. PMSCs treated with LY294002, a PI3K inhibitor upstream of AKT, under muscle differentiation conditions showed less skeletal muscle compaction at 7 days compared to muscle differentiation alone, but the addition of IGFBP-6 with LY294002 delayed these changes (more compaction) as seen with the white arrows compared to the inhibitor alone (20x). Images are representative of 3 independent experiments from one preterm placenta.

**Figure 5:**
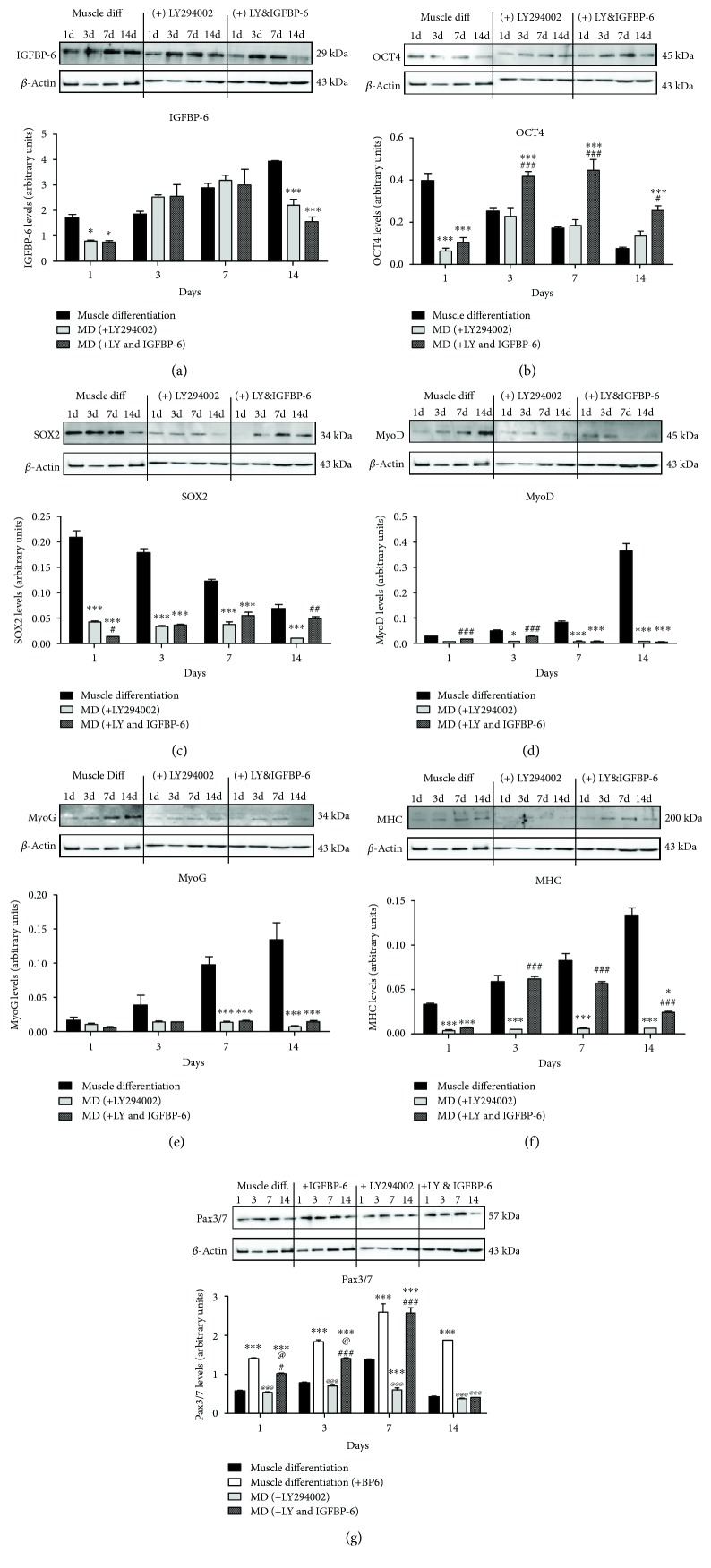
LY294002 treatment reduced IGFBP-6 and potency-associated and muscle differentiation markers. (a) IGFBP-6 protein levels decreased with LY294002 at days 1 and 14 as compared to muscle differentiation alone, and adding extracellular IGFBP-6 with the treatment did not cause additional changes to IGFBP-6 levels. (b, c) PMSCs treated with LY294002 decreased OCT4 (at day 1) and SOX2 (at each time point) compared to muscle differentiation, whereas IGFBP-6 addition with LY294002 increased OCT4 expression at 3, 7, and 14 days and at day 14 for SOX2 compared to LY294002 alone. Muscle lineage differentiation marker protein levels were reduced with LY294002 for (d) MyoD from 3 to 14 days, (e) MyoG at 7 and 14 days, and (f) MHC at all time points. IGFBP-6 supplementation with LY294002 increased MyoD levels at 1 and 3 days and MHC levels at 3, 7, and 14 days compared to LY294002 treatment. (g) Pax3/7 protein levels decreased with LY294002 at day 7. IGFBP-6 addition with the inhibitor increased Pax3/7 levels until day 7. Protein levels were quantified by densitometry and normalized to *β*-actin. Data is presented as the mean ± SEM of 3 independent experiments from one preterm placenta. Two-way ANOVA with Bonferroni's multiple comparison test was performed to determine ^∗^ *P* < 0.05, ^∗∗^ *P* < 0.01, and ^∗∗∗^ *P* < 0.001 compared to muscle differentiation conditions ^#^ *P* < 0.05, ^##^ *P* < 0.01, and ^###^ *P* < 0.001 compared to LY294002, or ^@@@^ *P* < 0.001 compared to muscle differentiation with IGFBP-6.

To better understand downstream signaling of the IGF-1R, LY294002 was used to inhibit PI3K signaling pathway. LY294002 alone reduced differentiated muscle morphology at 7 days (Figure 4 and Supplementary Figure 2). However, the addition of IGFBP-6 with LY294002 delayed these changes until day 14 postdifferentiation (Figure 4 and Supplementary Figure 2). Using immunoblotting, IGFBP-6 expression was decreased at days 1 and 14 in the presence of LY294002 and remained decreased despite IGFBP-6 supplementation (Figure 5(a)). Furthermore, LY294002 treatment reduced the protein levels of the potency-associated markers OCT4 (Figure 5(b)) at day 1 and SOX2 (Figure 5(c)) at all time points as compared to muscle differentiation alone. After IGFBP-6 supplementation with LY294002 treatment, OCT4 levels were maintained higher at all time points, while SOX2 expression was higher at day 14 compared to LY294002 treatment alone (Figures 5(b) and 5(c)). The levels of the muscle lineage markers MyoD, MyoG, and MHC decreased with LY294002 treatment as compared with muscle differentiation alone (Figures 5(d)–5(f)). IGFBP-6 addition with LY294002 treatment increased MyoD protein levels at days 1 and 3 (Figure 5(d)), while MHC levels were increased compared to LY294002 treatment alone (Figure 5(f)). These results indicated that muscle commitment occurred earlier in the presence of IGFBP-6 with LY294002 treatment. To confirm this hypothesis, Pax3/7, the muscle commitment marker, expression was tested. Pax3/7 protein levels were increased after IGFBP-6 addition with LY294002 (Figure 5(g)), suggesting an earlier commitment to the muscle lineage when IGFBP-6 was present.

**Figure 6:**
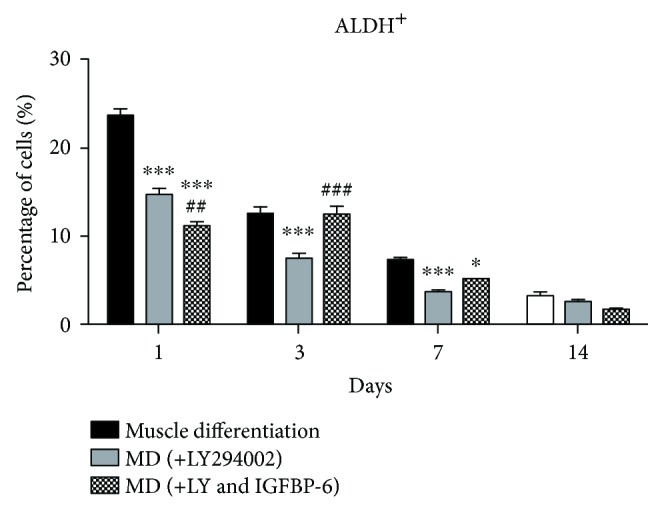
PMSCs treated with LY294002 and LY294002 with IGFBP-6 under skeletal muscle differentiation conditions decreased the frequency of cells with high ALDH activity. Compared to the PMSCs under muscle differentiation conditions, cells treated with LY294002 showed a decreased frequency of cells with high ALDH activity at 1, 3, and 7 days. IGFBP-6 with LY294002 treatment decreased frequency of cells with high ALDH activity only at day 1 and a significantly increased ALDH^+^ cells at day 3 compared to LY294002 treatment alone. Data is presented as the mean ± SEM of 3 independent experiments from one preterm placenta. Two-way ANOVA with Bonferroni's multiple comparison test was performed to determine ^∗^ *P* < 0.05 and ^∗∗∗^ *P* < 0.001 compared to muscle differentiation or ^##^ *P* < 0.01 and ^###^ *P* < 0.001 compared to LY294002 alone.

We used the Aldefluor™ assay to determine the frequency of progenitor cells with high ALDH activity, a more primitive progenitor phenotype. Compared to PMSCs under muscle differentiation alone, there was a decrease in the frequency of cells with high ALDH activity (ALDH^+^ cells) in PMSCs treated with LY294002 until day 7 (Figure 6 and Supplementary Figure 3). Moreover, IGFBP-6 with LY294002 treatment reduced the frequency of cells with high ALDH activity at day 1 but was maintained at a higher number compared to LY294002 alone at day 3. Thus, IGFBP-6 prolonged the progenitor phenotype in PMSCs when the PI3K pathway is inhibited under muscle differentiation conditions. These results show that the PI3K pathway is essential for muscle differentiation, and when the pathway was inhibited, IGFBP-6 could overcome the impact by allowing the cells to commit earlier to the muscle lineage and enhancing late-stage differentiation.

### 3.3. MAPK Signaling Is Required for PMSC Differentiation into the Skeletal Muscle

**Figure 7:**
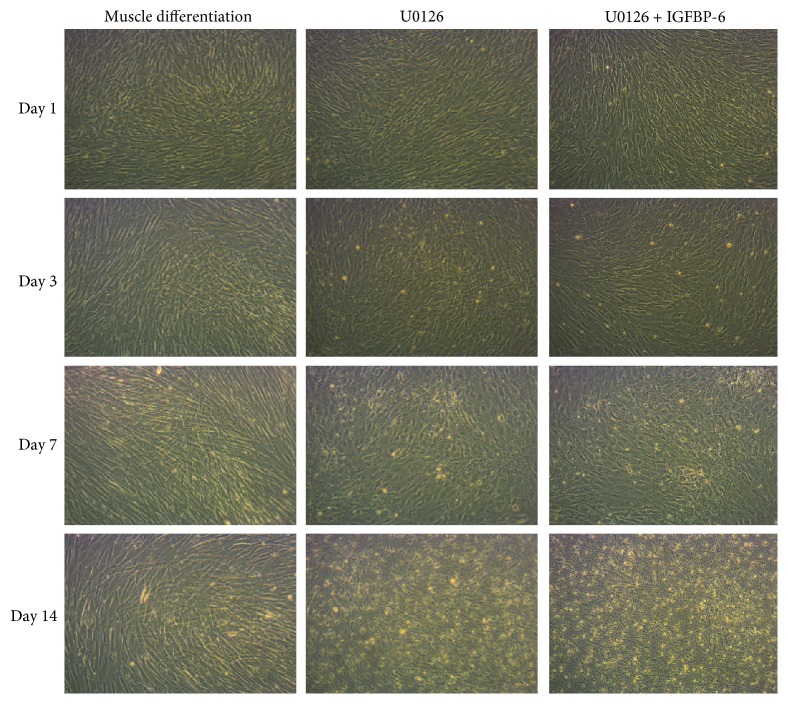
PMSCs treated with U0126 under muscle differentiation conditions showed reduced muscle compaction at 3 days. PMSCs under muscle differentiation conditions with U0126, a MEK inhibitor upstream of ERK1/2, showed less skeletal muscle differentiation at 3, 7, and 14 days with a change in cell morphology at 14 days compared to muscle differentiation (10x). The images are the representative of 3 independent experiments from one preterm placenta.

**Figure 8:**
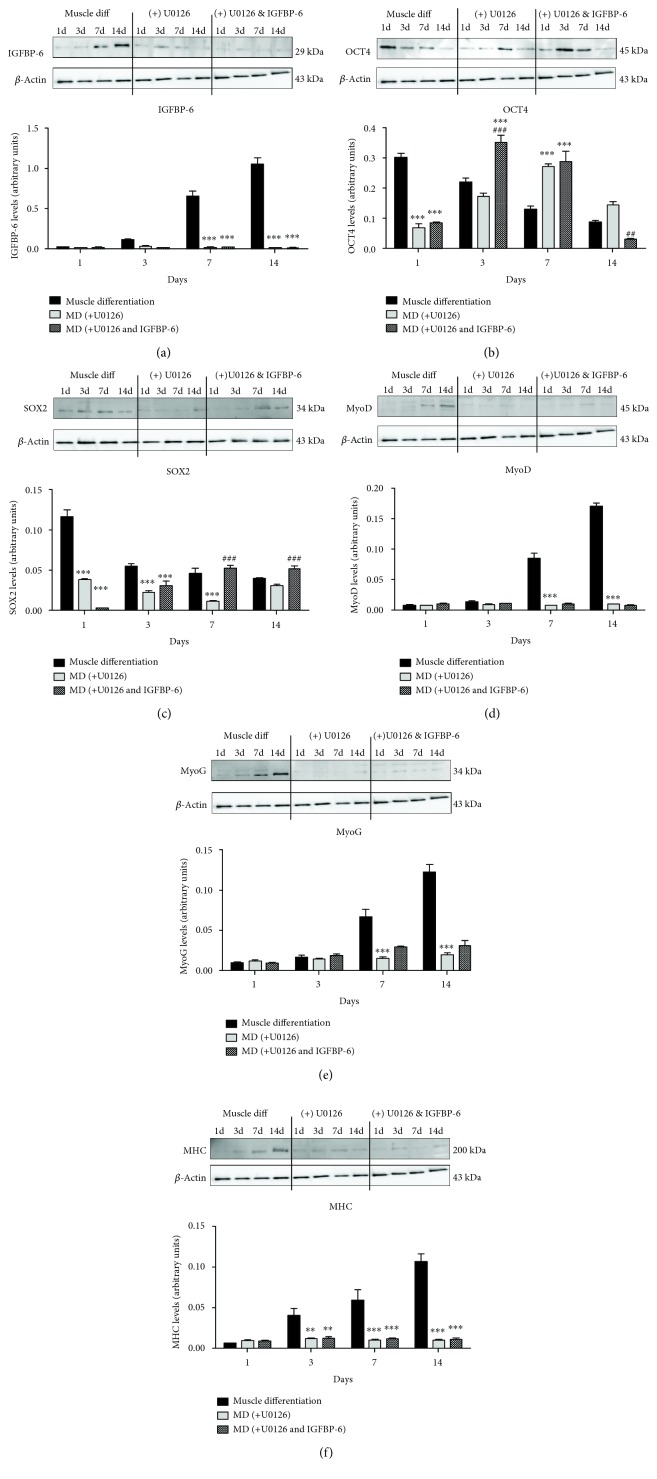
PMSCs treated with U0126 under muscle differentiation conditions reduced protein levels of IGFBP-6 and potency-associated and muscle differentiation markers. (a) U0126 decreased IGFBP-6 protein levels and adding IGFBP-6 with U0126 did not cause additional changes to IGFBP-6 levels. (b) Potency-associated marker OCT4 protein levels reduced with U0126 treatment at day 1with increased levels at 7 days. IGFBP-6 addition with U0126 increased OCT4 levels at 3 and 7 days compared to U0126 treatment alone. (c) SOX2 protein levels were lower at 1, 3, and 7 days compared to muscle differentiation. IGFBP-6 addition with U0126 decreased SOX2 levels at day 1 with an increase at 7 and 14 days compared to U0126. (d–f) Protein levels of the muscle differentiation markers MyoD, MyoG, and MHC were decreased at the later time points with U0126 and adding IGFBP-6 with U0126 did not change these effects. Protein levels were quantified by densitometry and normalized to *β*-actin. Data is presented as the mean ± SEM of 3 independent experiments from one preterm placenta. Two-way ANOVA with Bonferroni's multiple comparison test was performed to determine ^∗^ *P* < 0.05, ^∗∗^ *P* < 0.01, and ^∗∗∗^ *P* < 0.001 compared to muscle differentiation or ^#^ *P* < 0.05, ^##^ *P* < 0.01, and ^###^ *P* < 0.001 compared to U0126.

**Figure 9:**
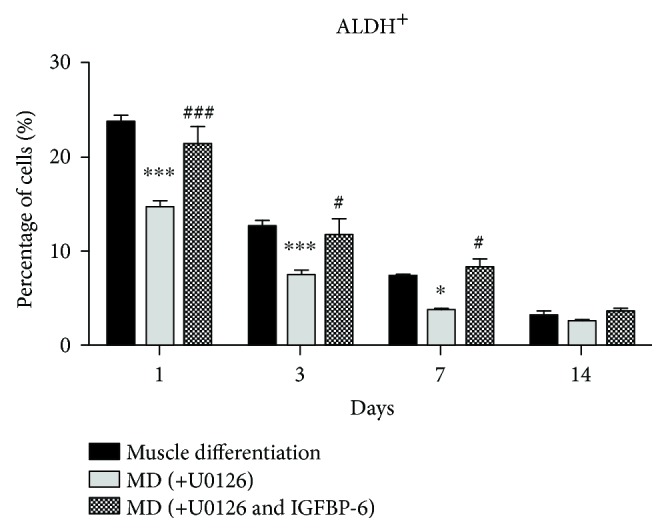
PMSCs treated with U0126 and U0126 with IGFBP-6 under skeletal muscle differentiation conditions decreased the frequency of cells with high ALDH activity. U0126 treatment showed decreased frequency of cells with high ALDH activity at 1, 3, and 7 days compared to muscle differentiation alone. IGFBP-6 supplementation with U0126 increased frequency of cells with high ALDH activity at 1, 3, and 7 days compared to U0126. Data is presented as the mean ± SEM of 3 independent experiments from one preterm placenta. Two-way ANOVA with Bonferroni's multiple comparison test was performed to determine ^∗^ *P* < 0.05 and ^∗∗∗^ *P* < 0.001 compared to muscle differentiation or ^##^ *P* < 0.01 and ^###^ *P* < 0.001 compared to U0126.

To test the downstream signaling of the IGF-1R *via* the MAPK pathway, U0126 was used to inhibit MAPK signaling, which phosphorylates ERK1/2. PMSCs treated with U0126 under muscle differentiation conditions showed reduced muscle cell compaction from 3 to 14 days with a change in muscle morphology compared to the PMSCs under muscle differentiation alone. IGFBP-6 supplementation with U0126 treatment showed similar morphology to U0126 alone (Figure 7). Using immunoblotting, IGFBP-6 levels were reduced with U0126 treatment and adding IGFBP-6 did not increase IGFBP-6 levels, indicating that MAPK is an important pathway for IGFBP-6 production (Figure 8(a)). OCT4 levels were reduced at day 1 by U0126 alone or U0126 with IGFBP-6 addition; however, U0126 with IGFBP-6 treatment maintained higher levels of OCT4 at 7 and 14 days until a significant decrease at day 14 compared to U0126 alone (Figure 8(b)). In contrast, potency-associated marker SOX2 protein levels were decreased by U0126 until 7 days and were increased by IGFBP-6 at 7 and 14 days compared to U0126 alone (Figure 8(c)). The protein levels of the early and late muscle lineage differentiation markers MyoD, MyoG, and MHC were significantly reduced after day 3 with U0126, and adding IGFBP-6 with U0126 did not reverse these effects (Figures 8(d)–8(f)). These findings suggest that MAPK is a critical pathway for PMSC skeletal muscle differentiation and cannot be substituted by an alternative pathway. The fact that IGFBP-6 did not accumulate in the intracellular environment when MAPK was inhibited shows that the MAPK pathway may be important for IGFBP-6 action on PMSC differentiation; however, this is not the only possible explanation, and it could be due to other effects caused by the MAPK inhibition or because the IGF-1R-dependent pathway is involved. PMSCs treated with U0126 under muscle differentiation conditions decreased the frequency of cells with high ALDH activity compared to the PMSCs under untreated muscle differentiation condition at 1, 3, and 7 days (Figure 9 and Supplementary Figure 4). In contrast, adding IGFBP-6 with U0126 treatment increased the frequency of cells with high ALDH activity compared to the PMSCs treated with U0126 alone. Therefore, in PMSCs under muscle differentiation conditions, IGFBP-6 acts in an IGF-1R-dependent manner mainly through the MAPK pathway.

Consequently, triggering downstream phosphorylation of AKT or ERK1/2 independent of IGF-1R activation by IGFBP-6 *via* an unknown mechanism could be responsible for IGFBP-6 impact on muscle cell differentiation.

### 3.4. Inhibition of Insulin Receptor Signaling Delayed PMSC Differentiation into the Skeletal Muscle and Adding IGFBP-6 Rescued the Effects

**Figure 10:**
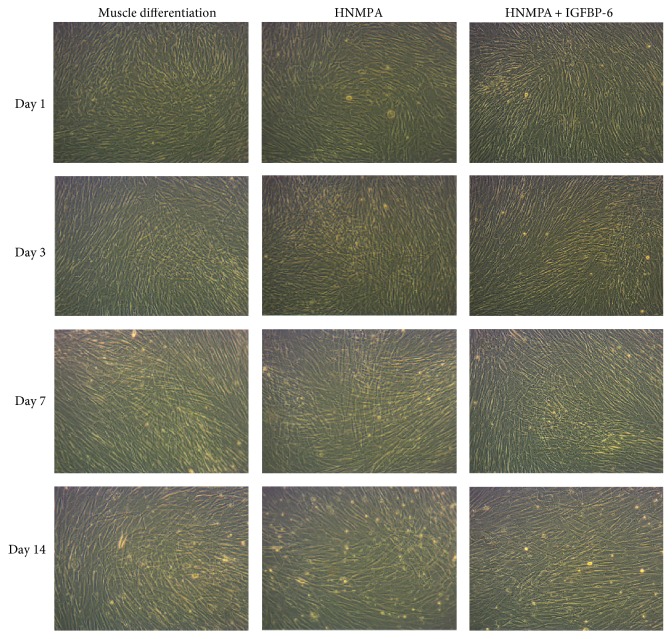
PMSCs under muscle differentiation conditions treated with HNMPA show delayed muscle compaction at 14 days. PMSCs under muscle differentiation conditions, with HNMPA or HNMPA with extracellular IGFBP-6, showed minimal change in skeletal muscle morphology and density at day 14 when compared to muscle differentiation (10x). The images are the representative of 3 independent experiments from one preterm placenta.

To test the role of insulin receptor (IR) signaling in the differentiation of PMSCs into the skeletal muscle, HNMPA was used to block IR kinase activity as it is specific for the IR and does not affect the IGF-1R. Neither HNMPA nor HNMPA with IGFBP-6 impacted differentiated cell morphology when compared to muscle differentiation conditions alone. However, HNMPA treatment delayed muscle differentiation (less compaction) at day 14, compared to control treatment (Figure 10 and Supplementary Figure 5).

**Figure 11:**
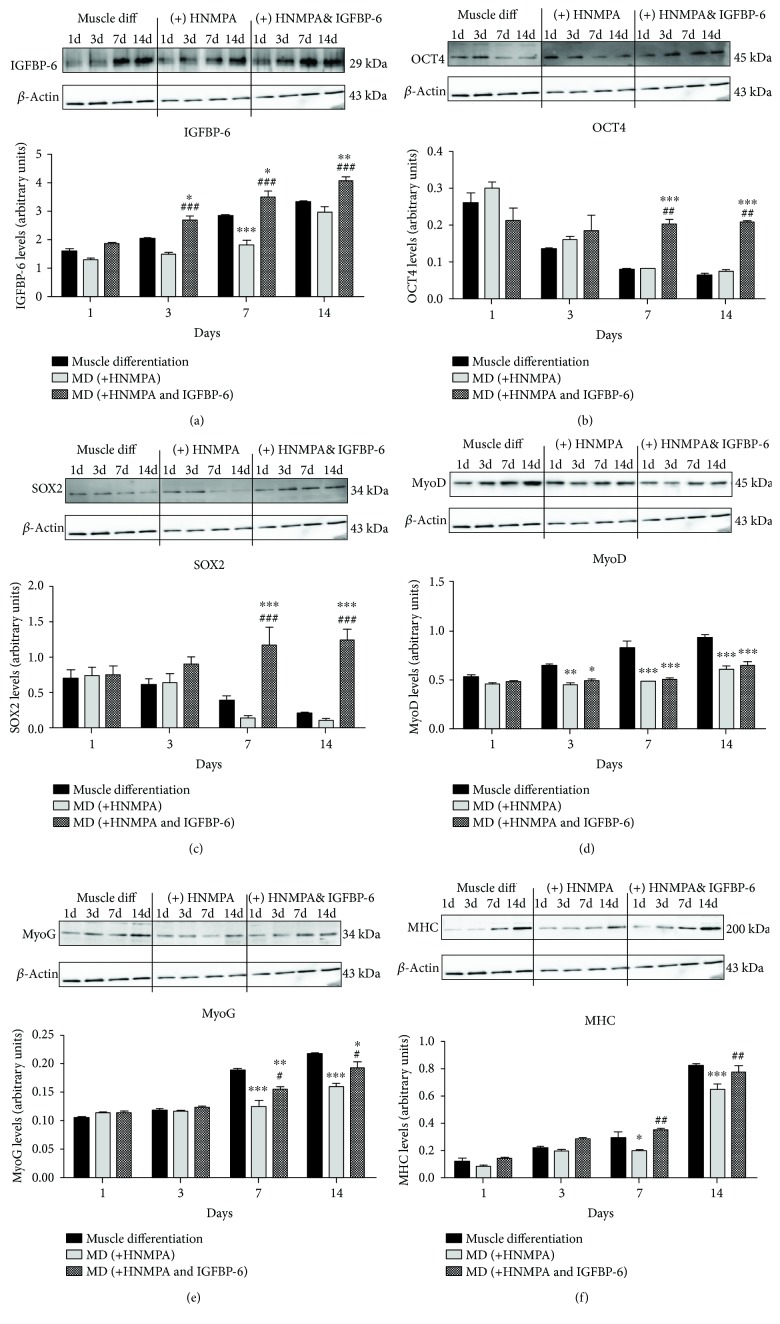
PMSCs treated with HNMPA under muscle differentiation conditions reduced muscle differentiation markers with no change in potency-associated markers. (a) HNMPA treatment decreased IGFBP-6 protein levels at 7 days as compared to muscle differentiation. IGFBP-6 supplementation with HNMPA increased IGFBP-6 levels at 3, 7, and 14 days. (b, c) HNMPA treatment did not change the protein levels of the pluripotency-associated markers OCT4 and SOX2. IGFBP-6 with HNMPA treatment increased the levels at 7 and 14 days. (d–f) HNMPA treatment decreased the protein levels of the muscle lineage differentiation markers MyoD, MyoG, and MHC at the later time points (3-14 days). IGFBP-6 addition with HNMPA treatment increased MyoG and MHC levels at 7 and 14 days compared to HNMPA treatment alone. Protein levels were quantified by densitometry and normalized to *β*-actin. Data is presented as the mean ± SEM of 3 independent experiments from one preterm placenta. Two-way ANOVA with Bonferroni's multiple comparison test was performed to determine ^∗^ *P* < 0.05, ^∗∗^ *P* < 0.01, and ^∗∗∗^ *P* < 0.001 compared to muscle differentiation conditions or ^#^ *P* < 0.05, ^##^ *P* < 0.01, and ^###^ *P* < 0.001 compared to HNMPA.

Intracellular IGFBP-6 levels were unchanged by HNMPA except for a reduction at day 7; however, adding IGFBP-6 with HNMPA increased IGFBP-6 protein levels at 3, 7, and 14 days (Figure 11(a)). HNPMA did not change the protein levels of the potency-associated markers (OCT4 and SOX2) but addition of extracellular IGFBP-6 with HNMPA increased both markers at days 7 and 14 compared to the PMSCs under muscle differentiation conditions and PMSCs treated with HNMPA (Figures 11(b) and 11(c)). Additionally, the levels of the muscle lineage differentiation markers MyoD, MyoG, and MHC were decreased at the later time points with HNMPA compared to PMSCs under muscle differentiation conditions, and extracellular IGFBP-6 increased MyoG and MHC levels at 7 and 14 days compared to HNMPA alone (Figures 11(d)–11(f)). These results suggest that insulin or IGFs could trigger myogenic differentiation; however, IGFBP-6 could also promote differentiation independent of insulin or IGFs.

## 4. Discussion

The promise of using stem cells in treating diseases is becoming closer to be used in the clinic [32, 33]. Still, understanding the niche factors and their influence on stem cell proliferation and differentiation *in vitro* is essential before stem cells can be used safely in regenerative medicine applications [34]. Muscle differentiation is a multistep process, starting with commitment to the muscle lineage and ending with the formation of multinucleated myotubes [2]. The IGF family is an essential early niche factor for stem cell survival, growth, proliferation, and differentiation [24]. It is also important in the skeletal muscle niche, with a major role in muscle development [5, 6, 9]. IGFBP-6 is expressed in the developing cells [23, 26–28]. We have demonstrated that the balance between intracellular and extracellular IGFBP-6 levels is required for modulating muscle differentiation by PMSCs [26] and that the effects of IGFBP-6 on muscle differentiation are both IGF-dependent and IGF-independent [31]. These findings provided basic insight into the role of IGFBP-6 and IGFs on PMSC muscle differentiation. The aim of this study was to characterize the effects of IGF-1R and IR activation on the differentiation of PMSCs into skeletal muscle and to investigate IGFBP-6 role in this process.

In these studies, we demonstrated that IGF-1R and its downstream signaling pathways (PI3K-AKT and MAPK pathways) were required for PMSC muscle differentiation. We also showed that when the PI3K pathway was inhibited, increased extracellular IGFBP-6 improved PMSC differentiation into the skeletal muscle as seen with the increased protein levels of MyoD and MHC. In contrast, MAPK pathway inhibition could not be rescued by increased extracellular IGFBP-6 as seen with the unchanged protein levels of the muscle lineage differentiation markers. MAPK inhibition also caused a significant decrease in intracellular IGFBP-6 concentrations, which could not be reversed by the addition of extracellular IGFBP-6 as seen with the unaffected IGFBP-6 protein levels. These studies suggested that MAPK signaling is an important pathway for PMSC differentiation into the skeletal muscle and that intracellular IGFBP-6 compliments this process. Therefore, we suggest that in PMSCs, IGFBP-6 acts in an IGF-1R-dependent manner predominantly through the MAPK signaling pathway and not through PI3K to achieve skeletal muscle differentiation. We further verified the importance of the insulin receptor (IR) in PMSC differentiation into the muscle and the interaction with IGFBP-6.

We demonstrated that IR plays an important role in PMSC muscle differentiation in addition to IGF-1R. We showed that inhibiting IR signaling delayed PMSC differentiation into the skeletal muscle but did not completely block the process as IGF-1R signaling was still active and most likely mediated the differentiation process. These observations also suggested that the induction of muscle differentiation by the high concentration of insulin (10 *μ*g/mL) in the media is likely exerted by insulin binding to the IGF-1R, to which it has low-affinity binding capacity. The fact that IGFBP-6 enhanced muscle differentiation when IR was inhibited suggests that IGFBP-6-induced PMSC differentiation into the muscle could occur independent of IR signaling.

The IGF-1R and IR are both receptor tyrosine kinases that activate several signaling transduction pathways [20, 21]. IGFs and insulin both promote cell proliferation and differentiation [10, 18–20], and IGFs also possess insulin-like metabolic effects, including increased glucose uptake in the skeletal muscle, mediated by either IGF-1R or IR [35]. Previous reports show that high concentrations of insulin activates both IGF-1R and IR [36, 37]; however, not much attention is given to IGF-1R binding affinity and effects versus IR when insulin is used.

Therefore, in future studies, a phosphokinase array may be used to specify interacting adaptors and signaling proteins within complementary IGF-1R and IR signaling pathways. Also, alterations in the PMSC microenvironment can cause epigenetic changes, and it will be interesting to understand whether the IGF system affects potency and myogenesis through epigenetic modulation of promoter regions.

These results are in agreement with previous reports on the importance of IGF-1R and its downstream pathways and the IR in muscle development and differentiation. However, this study is the first to show these effects on human stem cells isolated from the placenta and that IGFBP-6 addition enhanced the muscle differentiation process of PMSCs when IGF-1R or IR were inhibited *in vitro*.

Different signaling pathways, including IGF-1R and IR, crosstalk, and the complexity of signaling and its effects on PMSC differentiation into the muscle are beyond the scope of one study. The possibility that a different pathway, not examined in this study, is responsible for IGFBP-6 effects on PMSC differentiation into the skeletal muscle must be considered and further investigated to better understand the IGFBP-6 role in this differentiation process. Moreover, to confirm the results from this study, increasing the sample number to have biological replicates is vital as experiments were performed from one preterm placental tissue (15 weeks). Therefore, future studies are warranted to directly compare MSCs from the chorionic villi of different gestations (preterm and full-term human placentae), which will further improve our understanding of skeletal muscle differentiation and the effects of the IGF system based on ontogeny, and will help in choosing the best gestation age PMSC for skeletal muscle differentiation.

**Figure 12:**
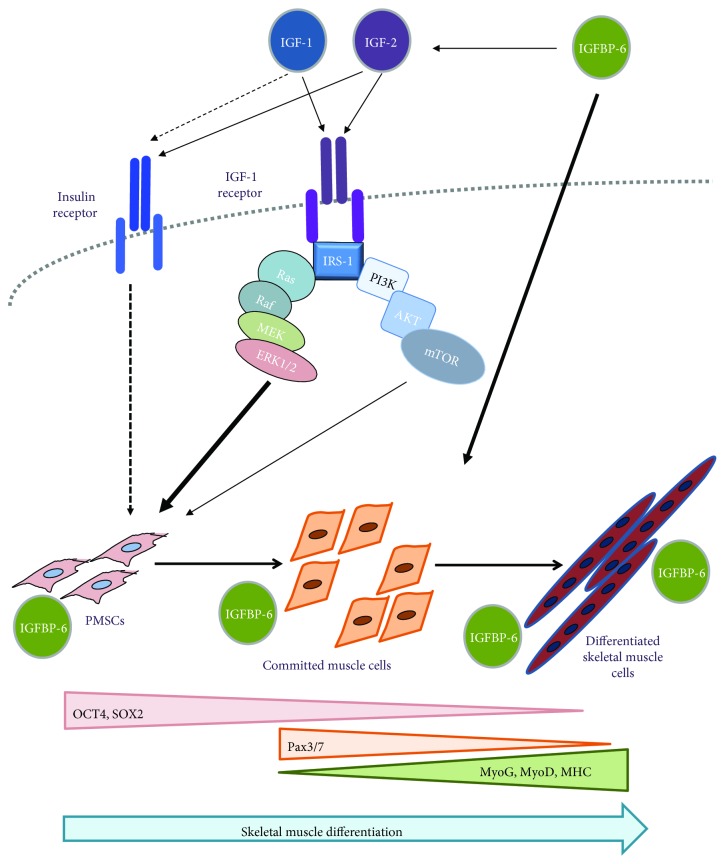
Schematic of the insulin-like growth factor system role in PMSC differentiation into the skeletal muscle. PMSCs isolated from the chorionic villus of preterm human placenta differentiated into the skeletal muscle under appropriate culture conditions. As PMSCs differentiated into the skeletal muscle, the levels of the potency-associated markers decreased, cells became committed to the muscle lineage, and skeletal muscle differentiation marker levels increased. IGFs bind to the IGF-1R and activate the tyrosine kinase activity to achieve muscle differentiation via downstream signaling pathway (PI3K-AKT and MAPK). The insulin receptor is also important in PMSC skeletal muscle differentiation. Moreover, IGFBP-6, due to its location, binds IGFs and enhances the muscle differentiation process through the IGF-1R or directly impacts PMSC muscle differentiation through IGF-independent functions. When IGF-1R or IR was inhibited *in vitro*, IGFBP-6 addition enhanced the muscle differentiation process of PMSCs with MAPK being a critical pathway for this differentiation process.

To date, previous studies on the role of the IGF family, specifically IGFBP-6, have not been reported during the differentiation of PMSCs towards the skeletal muscle lineage. Therefore, data presented in this study provides insight into the mechanisms of differentiation from PMSCs into the skeletal muscle by IGFs and IGFBP-6 during development and suggests that both the IGF-1R and IR signaling are important signaling pathways in PMSC differentiation towards skeletal muscle lineage. In addition, IGFBP-6 is also important for differentiation to occur, due to a combination of IGF-dependent and IGF-independent functions (Figure 12). Overall, manipulating the PMSC microenvironment using the IGF system, particularly IGFBP-6, can improve PMSC myogenic differentiation, a first step towards PMSC use for muscle regeneration therapies.

## Data Availability

The data used to support the findings of this study are available and included within the article and the supplementary information file.

## Supplementary Materials

Supplementary Materials Supplementary Figure 1: PMSCs under muscle differentiation conditions treated with different concentrations of PI3K, MAPK, and IR inhibitors. Cells were treated with different concentrations of (A) LY294002 (PI3K inhibitor), (B) U0126 (MAPK inhibitor), or (C) HNMAP (insulin receptor inhibitor) for 3 days under muscle differentiation conditions to assess the optimal concentration to be used. The concentration of the inhibitors was selected based on maintaining low band intensity for the duration of the experiment (3 days) compared to muscle differentiation alone. 25 *μ*M of the AKT inhibitor LY294002, 10 *μ*M of the ERK1/2 inhibitor U0126, and 10 *μ*M of the IR inhibitor HNMPA were selected. Protein levels were quantified by densitometry and normalized to total AKT, total ERK1/2, or *β*-actin. Data is presented as the mean ± SEM of 3 independent experiments from one preterm placenta. Two-way ANOVA with Bonferroni's multiple comparison test was performed to determine ^∗^ *P* < 0.05, ^∗∗^ *P* < 0.01, and ^∗∗∗^ *P* < 0.001 compared to muscle differentiation conditions. Supplementary Figure 2: PMSCs treated with LY294002, a PI3K inhibitor upstream of AKT, under muscle differentiation conditions showed less skeletal muscle morphology at 7 and 14 days, but the addition of IGFBP-6 with LY294002 delayed these changes until day 14 (10x). The images are the representative of 3 independent experiments from one preterm placenta. Supplementary Figure 3: representative flow cytometry dot plots showing the frequency of PMSCs with high ALDH activity when cultured under muscle differentiation conditions with or without LY294002 or LY294002 and IGFBP-6 at (A) day 1, (B) day 3, (C) day 7, and (D) day 14. DEAB-treated controls were used to establish the ALDH gate (data not shown). Supplementary Figure 4: representative flow cytometry dot plots showing the frequency of PMSCs with high ALDH activity when cultured under muscle differentiation conditions with or without either U0126 or U0126 and extracellular IGFBP-6 at (A) day 1, (B) day 3, (C) day 7, and (D) day 14. DEAB-treated controls were used to establish the ALDH gate (data not shown). Supplementary Figure 5: higher magnification of PMSCs treated with HNMPA or with IGFBP-6 supplementation with HNMPA. PMSCs treated with HNMPA under muscle differentiation conditions showed less skeletal muscle compaction and density at 14 days compared to muscle differentiation alone, but the addition of IGFBP-6 with HNMPA showed more muscle compaction as seen with the white arrows compared to HNMPA alone (20x). The images are the representative of 3 independent experiments from one preterm placenta.
