# Supplementary material for: Insulin-Like Growth Factor Binding Protein-6 Promotes the Differentiation of Placental Mesenchymal Stem Cells into Skeletal Muscle Independent of Insulin-Like Growth Factor Receptor-1 and Insulin Receptor
Source: Stem Cells Int. 2019 Feb 17;2019:9245938. doi: 10.1155/2019/9245938 (PMC6397983; doi:10.1155/2019/9245938)

Supplementary Figures

Supplementary Figure 1: PMSCs under muscle differentiation conditions treated with different concentrations of PI3K, MAPK, and IR inhibitors. Cells were treated with different concentrations of **(A)** LY294002 (PI3K inhibitor), **(B)** U0126 (MAPK inhibitor), or **(C)** HNMAP (insulin receptor inhibitor) for 3 days under muscle differentiation conditions to assess the optimal concentration to be used. The concentration of the inhibitors was selected based on maintaining low band intensity for the duration of the experiment (3 days) compared to muscle differentiation alone. 25 μM of the AKT inhibitor LY294002, 10 μM of the ERK1/2 inhibitor U0126, and 10 μM of the IR inhibitor HNMPA were selected. Protein levels were quantified by densitometry and normalized to total AKT, total ERK1/2, or β-Actin. Data is presented as the mean ± SEM of 3 independent experiments from one preterm placenta. Two-way ANOVA with Bonferroni's multiple comparison test was performed to determine $*P<0.05$, $**P<0.01$, $***P<0.001$ compared to muscle differentiation conditions.

Supplementary Figure 2: PMSCs treated with LY294002, a PI3K inhibitor upstream of AKT, under muscle differentiation conditions showed less skeletal muscle morphology at 7 and 14 days; but the addition of IGFBP-6 with LY294002 delayed these changes until day 14 (10X). Images are representative of 3 independent experiments from one preterm placenta.

Supplementary Figure 3: Representative flow cytometry dot plots showing the frequency of PMSCs with high ALDH-activity when cultured under muscle differentiation conditions with or without LY294002 or LY294002 and IGFBP-6 at **(A)** day 1, **(B)** day 3, **(C)** day 7, and **(D)** day 14. DEAB treated controls were used to establish the ALDH gate (data not shown).

Supplementary Figure 4: Representative flow cytometry dot plots showing the frequency of PMSCs with high ALDH-activity when cultured under muscle differentiation conditions with or without either U0126 or U0126 and extracellular IGFBP-6 at **(A)** day 1, **(B)** day 3 , **(C)** day 7 , and **(D)** day 14. DEAB treated controls were used to establish the ALDH gate (data not shown).

Supplementary Figure 5: Higher magnification of PMSCs treated with HNMPA or with IGFBP-6 supplementation with HNMPA. PMSCs treated with HNMPA under muscle differentiation conditions showed less skeletal muscle compaction and density at 14 days compared to muscle differentiation alone; but the addition of IGFBP-6 with HNMPA showed more muscle compaction as seen with the white arrows compared to HNMPA alone (20X). Images are representative of 3 independent experiments from one preterm placenta.

Supplementary Figure 1:

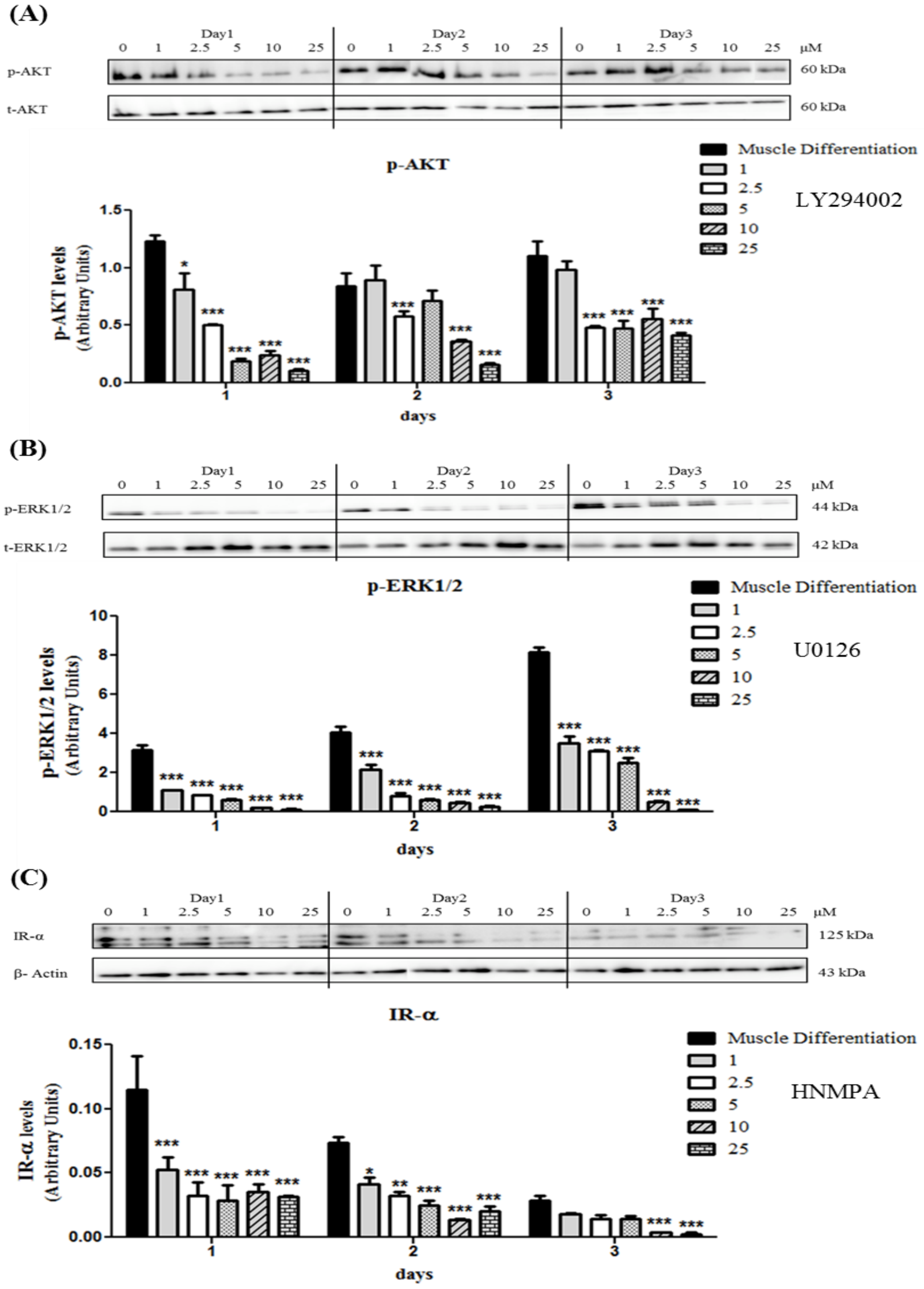

Supplementary Figure 2:

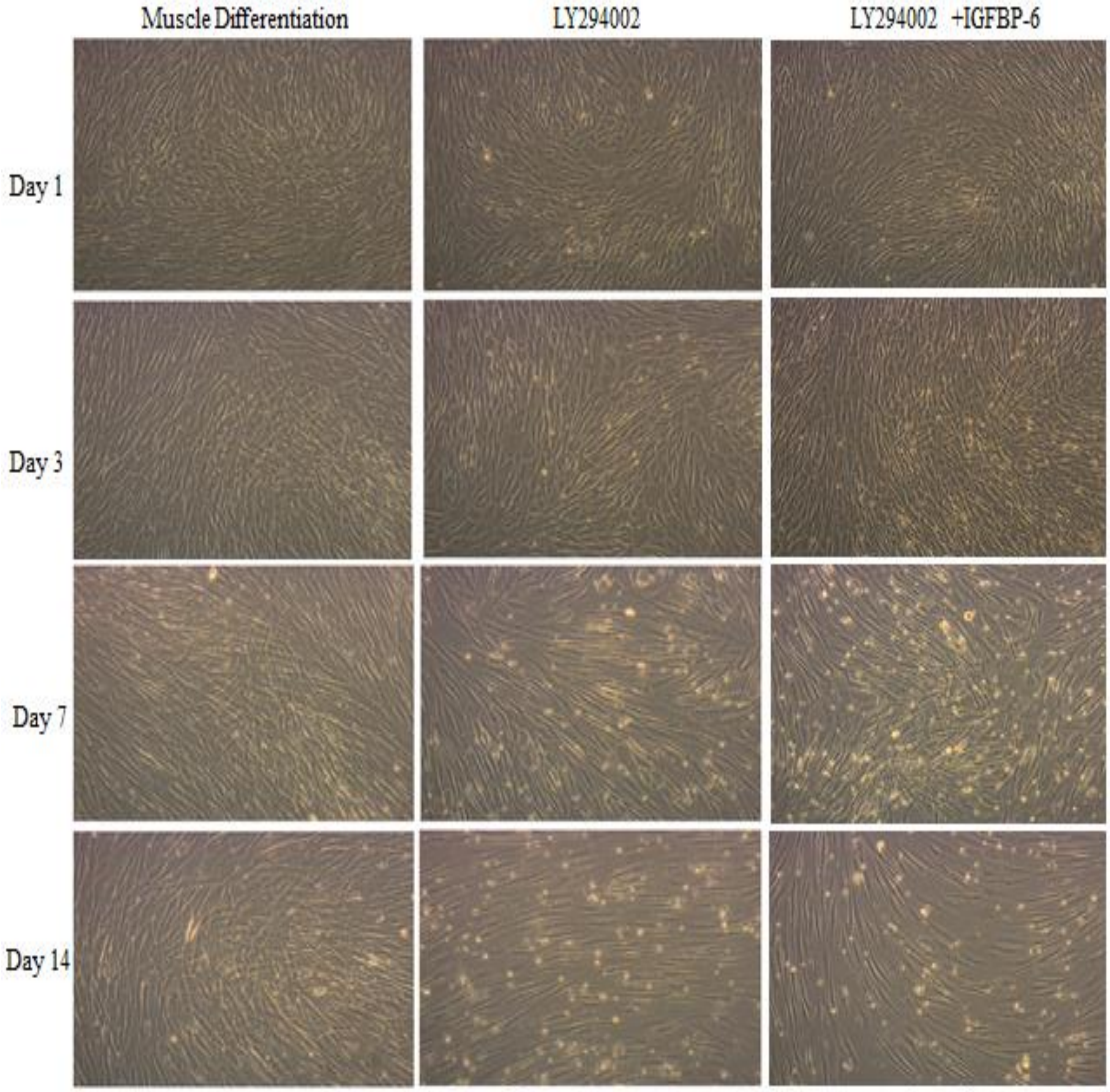

Supplementary Figure 3:

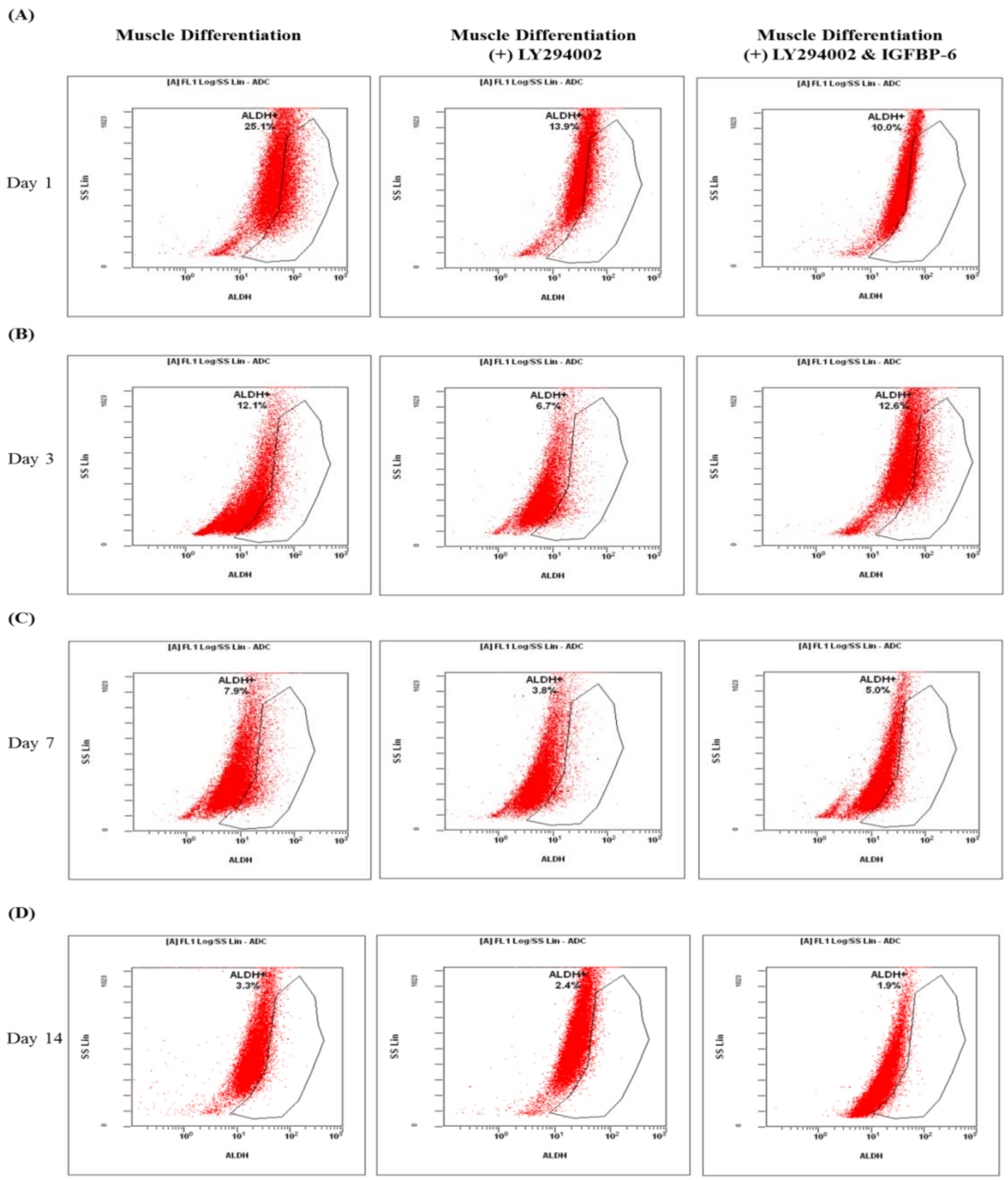

Supplementary Figure 4:

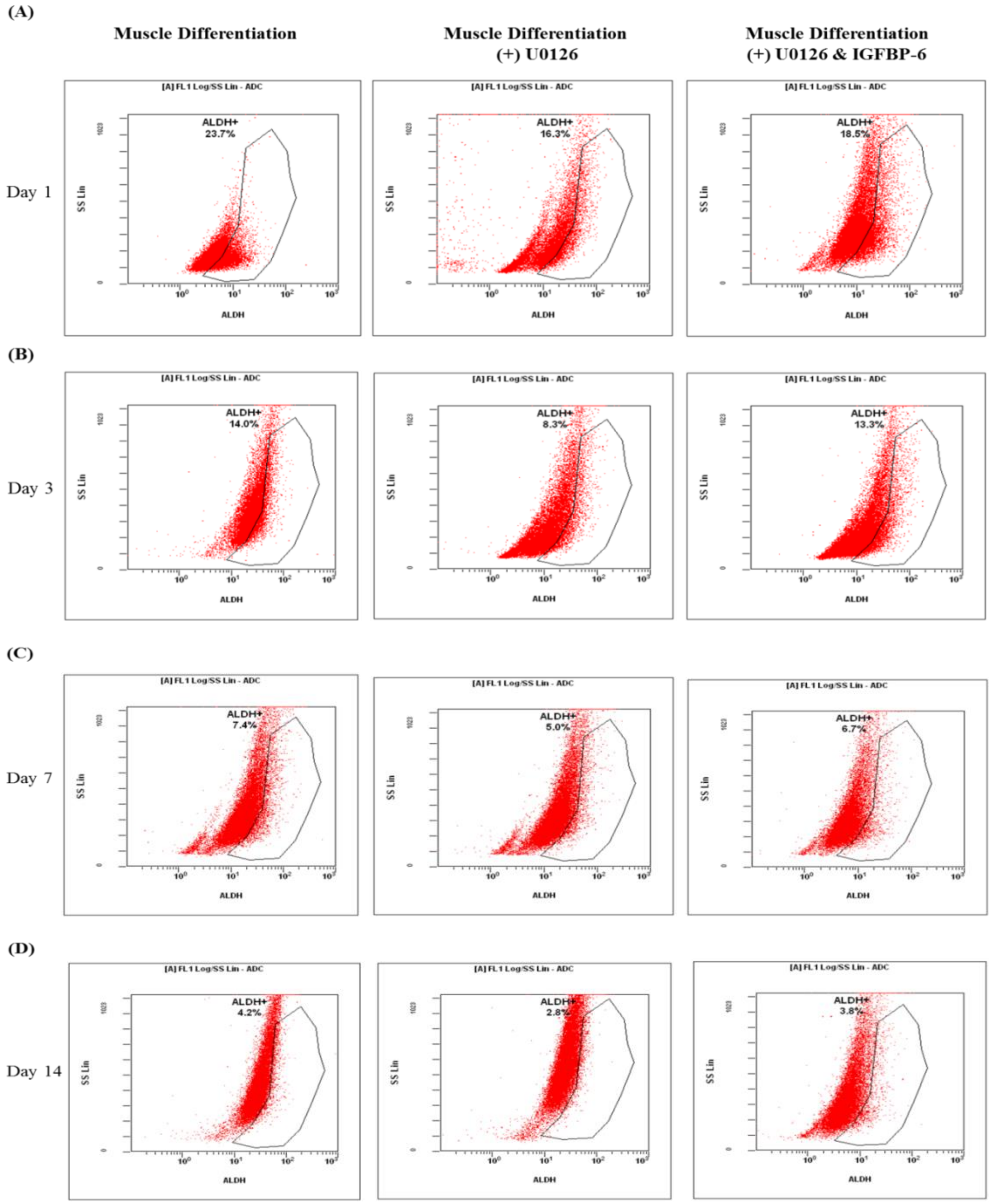

Supplementary Figure 5:

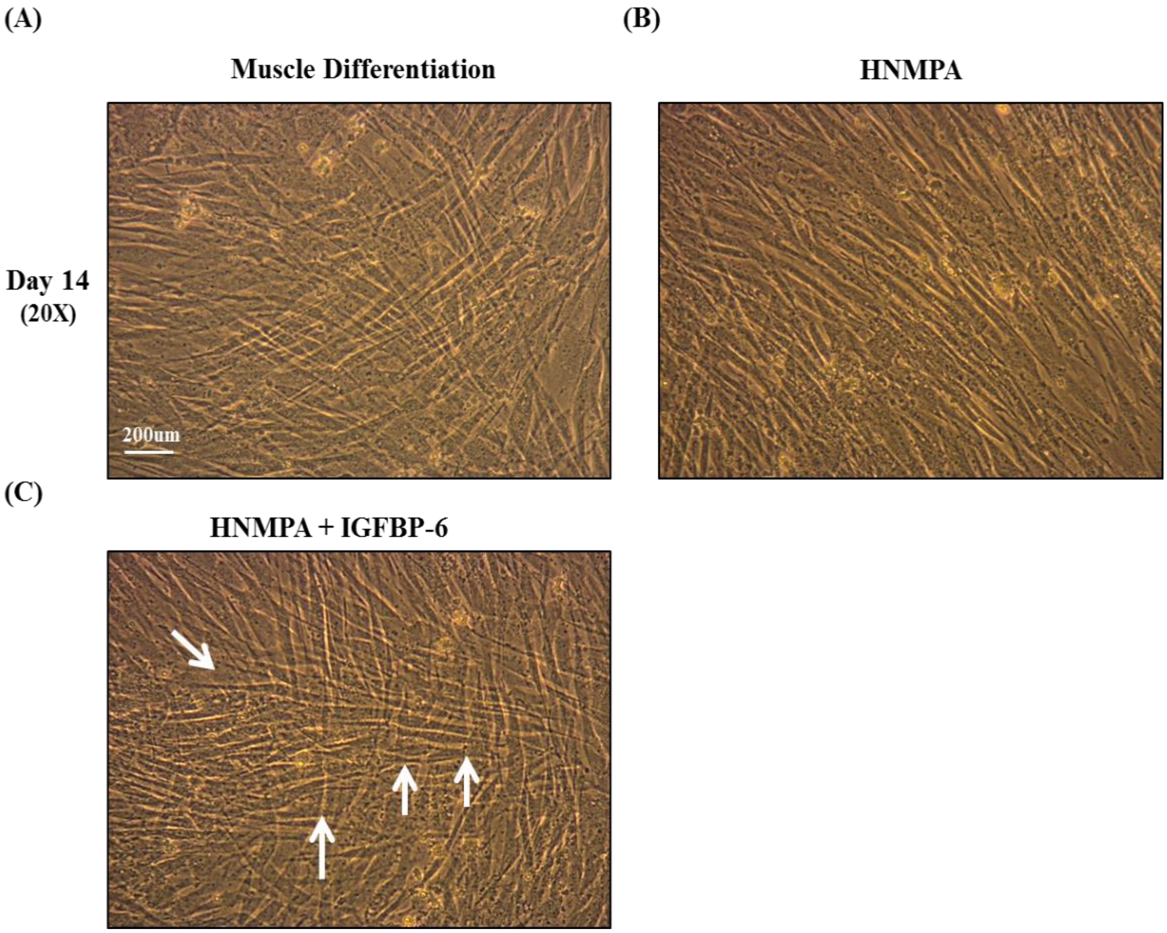

Supplement: Supplementary Materials — Supplementary Figure 1: PMSCs under muscle differentiation conditions treated with different concentrations of PI3K, MAPK, and IR inhibitors. Cells were treated with different concentrations of (A) LY294002 (PI3K inhibitor), (B) U0126 (MAPK inhibitor), or (C) HNMAP (insulin receptor inhibitor) for 3 days under muscle differentiation conditions to assess the optimal concentration to be used. The concentration of the inhibitors was selected based on maintaining low band intensity for the duration of the experiment (3 days) compared to muscle differentiation alone. 25 μM of the AKT inhibitor LY294002, 10 μM of the ERK1/2 inhibitor U0126, and 10 μM of the IR inhibitor HNMPA were selected. Protein levels were quantified by densitometry and normalized to total AKT, total ERK1/2, or β-actin. Data is presented as the mean ± SEM of 3 independent experiments from one preterm placenta. Two-way ANOVA with Bonferroni's multiple comparison test was performed to determine ∗ P < 0.05, ∗∗ P < 0.01, and ∗∗∗ P < 0.001 compared to muscle differentiation conditions. Supplementary Figure 2: PMSCs treated with LY294002, a PI3K inhibitor upstream of AKT, under muscle differentiation conditions showed less skeletal muscle morphology at 7 and 14 days, but the addition of IGFBP-6 with LY294002 delayed these changes until day 14 (10x). The images are the representative of 3 independent experiments from one preterm placenta. Supplementary Figure 3: representative flow cytometry dot plots showing the frequency of PMSCs with high ALDH activity when cultured under muscle differentiation conditions with or without LY294002 or LY294002 and IGFBP-6 at (A) day 1, (B) day 3, (C) day 7, and (D) day 14. DEAB-treated controls were used to establish the ALDH gate (data not shown). Supplementary Figure 4: representative flow cytometry dot plots showing the frequency of PMSCs with high ALDH activity when cultured under muscle differentiation conditions with or without either U0126 or U0126 and extracellular [file 9245938.f1.pdf]
